# Evaluation of an early detection tool for social-emotional and behavioral problems in toddlers: The Brief Infant Toddler Social and Emotional Assessment - A cluster randomized trial

**DOI:** 10.1186/1471-2458-11-494

**Published:** 2011-06-24

**Authors:** Ingrid Kruizinga, Wilma Jansen, Alice S Carter, Hein Raat

**Affiliations:** 1 Department of Public Health, Erasmus MC - University Medical Centre Rotterdam, PO BOX 2040, 3000 CA Rotterdam, the Netherlands; 2 Public Health Service and Environs, Rotterdam, PO BOX 70032, 3000 LP Rotterdam, The Netherlands; 3 Department of Psychology, University of Massachusetts Boston, 100 Morrissey Boulevard, Boston, MA 02125, USA

## Abstract

**Background:**

The prevalence of social-emotional and behavioral problems is estimated to be 8 to 9% among preschool children. Effective early detection tools are needed to promote the provision of adequate care at an early stage. The Brief Infant-Toddler Social and Emotional Assessment (BITSEA) was developed for this purpose. This study evaluates the effectiveness of the BITSEA to enhance social-emotional and behavioral health of preschool children.

**Methods and Design:**

A cluster randomized controlled trial is set up in youth health care centers in the larger Rotterdam area in the Netherlands, to evaluate the BITSEA. The 31 youth health care centers are randomly allocated to either the control group or the intervention group. The intervention group uses the scores on the BITSEA and cut-off points to evaluate a child's social-emotional and behavioral health and to decide whether or not the child should be referred. The control group provides care as usual, which involves administering a questionnaire that structures the conversation between child health professionals and parents. At a one year follow-up measurement the social-emotional and behavioral health of all children included in the study population will be evaluated.

**Discussion:**

It is hypothesized that better results will be found, in terms of social-emotional and behavioral health in the intervention group, compared to the control group, due to more adequate early detection, referral and more appropriate and timely care.

**Trial registration:**

Current Controlled Trials NTR2035

## Background

Psychosocial problems, such as social-emotional and behavioral problems, are prevalent among preschool children; in approximately 8-9 percent of preschool children, child health professionals identify psychosocial problems, such as anxious or depressed feelings and aggressive or disobedient behavior [1,2]. Psychosocial problems in this age group can interfere with everyday functioning [3-5]. Research has demonstrated that problems at preschool age are associated with depressive symptoms, oppositional defiant or conduct disorder, poor peer relationships and social skills, parent- and teacher reported problems with externalizing and internalizing behavior, poor academic performance and psychiatric problems later in life [6-9]. A retrospective study [10] demonstrated that adolescents with psychosocial problems already had neurocognitive, temperament and behavioral problems at age two or three years old.

It has been recommended that psychosocial problems can be detected at a very young age and followed by appropriate management [11-13]. Research has shown that detection and treatment of psychosocial problems at a young age significantly reduces problems and increases competencies [14,15]. Preventive youth health care, as part of community care or paediatrics, offers an opportunity for the early detection of psychosocial problems among preschool children. Child health professionals, such as physicians and nurses who provide preventive care, may apply early detection of psychosocial problems and if necessary adequate referral or short counselling [16]. In the Netherlands, participation of parents with their child in the preventive youth health care is free of charge and on voluntary basis; almost 95% of the parents of preschool children make use of the youth health care service [17].

Despite the potential impact of psychosocial problems at preschool age and the presence of easily accessible youth health care, only a relatively small number of children with psychosocial problems receive appropriate care [2,5]. One study showed that only 29% of the children with severe problems, based on a Child Behavior Checklist (CBCL) total problem score in the clinical range, were identified by child health professionals [2]. And, in another study, only 13% of the children who scored in the clinical range of the CBCL total problem score were referred to mental health services [5].

In current preventive youth health care in the Netherlands, child health professionals apply a structured questionnaire about psychosocial problems that parents complete before coming to the youth health care center, which helps to structure the conversation between child health professionals and parents; there are no validated norm tables or cut-off scores that indicate when the questionnaire signals a problem [18].

As an alternative to this procedure, it has been recommend to evaluate the use of the Brief Infant-Toddler Social and Emotional Assessment (BITSEA) [19] for 1-3 year olds, to detect children at-risk for psychosocial problems and to act upon detection in a coherent, effective way [20].

## Objectives

The objective of this study is to evaluate the effectiveness of the BITSEA as an early detection tool for preventive youth health care on children's psychosocial health at one year follow-up, compared to 'care as usual'. Additionally, the feasibility will be evaluated. In this paper we describe the design of this study.

## Methods and Design

### Study design

**Figure 1:**
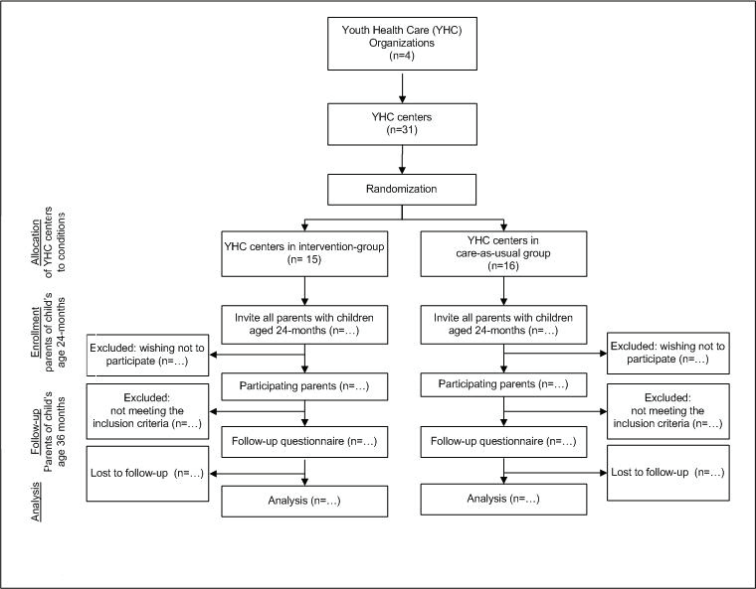
**Flow chart of the participants and allocation through the trial**.

The design of the study is a cluster randomized controlled trial in which parents of children aged about 24 months are invited to participate. Information on the study is provided to the parents and the parents are asked to provide informed consent. The parents/children are invited by preventive youth health care staff for a regular health check. We identified 31 distinct youth health care centers that were numbered to the purpose of randomization. We use a block randomization so that within each of the 4 organisations, youth health care centers were randomly allocated to either the control group or the intervention group, using random numbers. The child health professionals in the intervention group use the scores on the BITSEA and the cut-off points to assess whether children are at risk for psychosocial problems. The child health professionals in the control group offer usual care by children using a questionnaire for structuring the conversation with the parents. The effect of the intervention will be evaluated after one year of follow-up by comparing CBCL1,5-5 [21] scores between the children in the intervention group and children in the control group, taking into account the baseline measurement on the CBCL1,5-5. The course of the study is presented in Figure 1. The Medical Ethics Committee of the Erasmus Medical Centre Rotterdam approved the study protocol (reference number MEC02009-092).

### Study procedure and participants

#### Youth health care centers and randomisation procedure

Four youth health care organizations that consist of a total of 31 distinct youth health care centers that cover both urban and rural areas in the larger Rotterdam area, are participating in this study. Fifteen youth health care centers were randomly allocated to the intervention group; 16 youth health care centers were randomly allocated to the care-as-usual group, applying a block randomisation procedure as described above. Prior to the start of the study, the researchers arranged meetings to explain the study procedure and to instruct the child health professionals in the intervention group about the use and scoring of the BITSEA, with support of a specialized psychiatrist.

#### Children and their parents

Parents of 3,000 children are invited to participate in the study. The study population consists of parents or caregivers of toddlers aged 24 months old at baseline, and 36 months old at one year follow-up. Parents of children in the age range of this study have a high attendance (95%) at the regular health checks at youth health care centers [17]. Children who receive treatment of a mental health professional at baseline will be excluded from the study.

### Intervention condition

#### BITSEA

The 42-item BITSEA is an early detection tool for emotional or behavioral problems and delays in social-emotional competence, including autism spectrum disorders, in toddlers. The BITSEA was developed and applied in the USA, and since then also studied in Turkey and Finland [19,22,23]. It is appropriate for use among children of 12-36 months old and consists of 42 items with 3 response options ('not true/rarely', somewhat true/sometimes', 'very true/often'). That are part of one of 2 multi-item scales, a Problem scale (31 items) or Competence scale (11 items). Per scale the items are summed up into a scale score. In addition to the 42 items, the BITSEA has 2 single-item questions with regard to parents' concerns. Internal consistency of the Problem scale has been reported to be 0.79 and of the Competence scale 0.65 [19]. Ten to 45 day test-retest reliability (ICC) of the Problem scale has been reported to be 0.87 and of the Competence scale 0.85 [19]. Relative to typical parent/teacher agreement [24], the parent/child-care provider correlation was relatively high for the Competence scale (ICC = 0.59) and 0.28 for the Problem scale [19]. The BITSEA was translated into Dutch according to international guidelines [25]. Psychometric properties of the BITSEA for the Dutch population will be assessed in the present study.

At the intervention centers child health professionals use the BITSEA [26] as an early detection tool during the regular health check. The child health professionals are trained to score the answers given by parents on the BITSEA and use the cut-off points provided in the literature [19] in their assessment whether children are at risk for or currently experiencing psychosocial problems. Additional information given by parents about timing, duration, intensity of problematic emotions and behavior is also considered in the assessment of the risk for problems. If, for instance, the problematic emotions/behaviors are mild or are considered to be temporarily, e.g. after a major life event, the child health professional may offer advice about how to cope with the circumstances and may choose to ask the parent back in a few weeks for a follow-up.

The child health professional may choose to refer a child and his/her parents to specialized care when the child is at risk for or currently experiencing problems based on the BITSEA scores and cut-off points, when the problematic behaviors/emotions are severe and not considered to be temporarily. A referral to specialized care is always made after consultation with the physician at the youth health care center.

### Control condition

In the control condition youth health care centers at child's age 2 years provide care as usual; i.e. the child health professionals use a short questionnaire [18] that serves as a guide through the conversation between child health professionals and parents. Based on this information the child health professionals may choose to invite parents back for a follow-up visit or to refer to specialized care after consultation with the physician at the youth health care center.

## Measurements

### Primary outcome measures

The primary outcome of the study is the children's psychosocial health, measured with the Child Behavioral Checklist (CBCL1,5-5) [21]. Child health professionals are blind to this measurement. The 99-item CBCL1,5-5 is designed for children 18 months through 5 years and has two domains (Internalizing and Externalizing Problems and a Total Problem score). Answers are given on a 3-point scale with the following response options: 'not true', 'somewhat or sometimes true' and 'very true or often true'. We apply subclinical and clinical cutpoints for the Dutch population [27]. The primary outcome will be measured at baseline at child's age 24 months and one year after the intervention, at the child's age of 36 months. We hypothesize that children in the intervention group will have a lower Total Problem score on the CBCL1,5-5 at follow-up compared to children in the control group, due to more adequate screening, referral and more appropriate and timely care. For an overview, see table 1.

**Table 1:** Primary and secondary outcome measures and co-variates in the study

Primary outcome measure	Secondary outcome measure	Co-variates
-CBCL1,5-5**^b, f^**	-ITQOL**^f^**	-Date of birth**^b ^**(parents & child)
*(Total Problem score)*	*(General Health Perceptions subscale)*	- Sex**^b ^**(child)
	*(Growth and Development subscale)*	-Ethnicity**^b ^**(parents & child)
		-Immigration characteristics**^b ^**(parents)
		-Cultural identity**^b ^**(parents)
		-Social economical status**^b ^**(parents)
		-Day-care attendance**^b ^**(child)
		-Household composition**^b, f^**
		-Major life events**^b, f ^**(parents & child)
		-Presence of (mental) health****problems and treatment for those problems (child)**^b, f^**
		-Perceived health of the child rated by parents**^f^**

^b ^= measured at baseline (child's age 24 months),

^f ^= measured at 1 year follow-up (child's age 36 months)

### Secondary outcome measures

A secondary outcome is health related quality of life, i.e. General Health Perceptions subscale and the Growth and Development subscale of the Infant and Toddler Quality of Life Questionnaire (ITQOL) [28,29], measured at follow-up at child's age 36 months. For an overview, see table 1.

### Co-variates

Information on parental characteristics (date of birth, ethnicity, immigration characteristics, cultural identity, socio-economic status), children's characteristics (date of birth, sex, ethnicity, day-care attendance, presence of (mental) health problems and treatment for those problems), and household composition, major life events and the perceived health of the child rated by parents are obtained from the questionnaires at baseline and at follow-up. For an overview, see table 1.

### Statistical analyses

Given the cluster design of the study, multilevel analyses will be applied [30,31]. Linear multilevel analysis will be applied for continuous outcome variables and logistic multilevel analysis for dichotomous outcome variables. Interaction effects of gender and ethnic background with the outcomes will be explored.

### Power of the study

Power calculations indicated that a total of 3,000 children (and their parents) are needed to detect a difference of 8 points on the CBCL1,5-5 between the control and experimental group, assuming a standard deviation of the CBCL1,5-5 of 26.5 points [32] and an intra-cluster coefficient of 0.1, with a power of 0.80 and alpha 0.05. Assuming a participation of 50% and a lost to follow-up of 30%, we will have complete data at follow-up of 2,100 children (1,050 in both the intervention and the control group).

### Process evaluation

In addition to the effect evaluation, a process evaluation will be carried out, in which both the perspectives of parents and professionals will be taken into account. All parents that are included in this study, are asked to evaluate the use of the early detection tool (i.e. level of difficulty, level of understanding, consumed time and satisfaction with the early detection tool as a preparation for the regular health check at the youth health care center). All child health professionals are invited to complete a computer-based process evaluation questionnaire at 6 months after the start of the study. The process evaluation questionnaire consists of items about consumed time, adherence to work instructions, satisfaction with the early detection tool, general perception of the use of the early detection tools in the youth health care and perceived contribution of the early detection tool (a) to the quality of the conversation with parents, (b) to the assessment of the development of the child, and (c) to deciding whether or not to refer.

Furthermore, referrals and consumed care in the year after baseline measurement are assessed at 1-year follow-up, at child's age 36 months; i.e. if a referral to specialized care is made and to which professional; the extent to which parents pursue received referrals, and the diagnosis if one is made are measured at the 1 year follow-up, when children are age 36 months.

## Discussion

This paper describes the design of a cluster randomized controlled trial. The trial evaluates the effectiveness of the BITSEA as an early detection tool when used by preventive child health professionals on children's psychosocial health at one year follow-up, compared to 'care as usual'. We hypothesize to find better results, in terms of psychosocial health in the intervention group at one year follow-up, compared to the control group, due to more adequate early detection, referral and more appropriate and timely care.

Strengths of the study are the cluster randomized controlled design, the power of the study, and the setting of the study, which is the daily practice of regular health checks at the youth health care centers that are highly attended by parents. The one year follow-up measurement allows evaluation of the medium term effect of the BITSEA. The study sample will include families with a non-Dutch background, which we expect will add to the generalizability of the results.

Because the study relies primarily on self-report by parents, misclassification might occur. Parents might provide socially desirable answers, e.g. by understating problems or overstating competencies.

A limitation of the study is that the questionnaires are only available in Dutch. For this reason it might be possible that parents with a relatively low level of knowledge of the Dutch language will have some difficulty with the completion of the early detection tool. However, parents have the opportunity to ask for help regarding this issue at the youth health care centers. Furthermore we assess the extent in which parents have understood the questions in the early detection tool as a process measure.

In conclusion, the study evaluates the effectiveness of the BITSEA as an early detection tool to be applied by child health professionals, with the purpose of promoting children's psychosocial health at one year follow-up, compared to 'care as usual'.

## Competing interests

AC gets royalties on the sale of the BITSEA from Pearson Assessment (not in the context of this study). The other authors declare that they have no competing interests.

## Authors' contributions

HR and WJ had the original idea for the study and its design and were responsible for acquiring the study grant. IK is responsible for the data collection, data analysis en reporting of the study results. AC provides expert input during the study. HR and WJ supervise the study. All authors regularly participated in discussing the design and protocols used in the study. All authors read and approved the final manuscript.

## Pre-publication history

The pre-publication history for this paper can be accessed here:

http://www.biomedcentral.com/1471-2458/11/494/prepub
